# Supplementary material for: The king’s spice cabinet–Plant remains from Gribshunden, a 15th century royal shipwreck in the Baltic Sea
Source: PLoS One. 2023 Jan 26;18(1):e0281010. doi: 10.1371/journal.pone.0281010 (PMC9879437; doi:10.1371/journal.pone.0281010)
Supplement: Attachment — Submitted filename: Response to Reviewers.docx [file pone.0281010.s002.docx]

**Response to Reviewers**

# **Title: *The king’s spice cabinet – Plant remains from Gribshunden, a 15^th^ century royal shipwreck in the Baltic Sea***

We are grateful for the comments and suggestions by the reviewers and appreciate the time taken to do so. The suggestions have been very useful and have much improved the submitted manuscript. We have followed most of the recommendations and suggestions by the reviewers. All changes can be tracked in the Revised Manuscript with Track Changes.

We look forward to hearing from you regarding our submission. We will respond to any further questions and comments that you may have.

Thanks for your time!

Mikael

**Author comments on revision**

Specific corrections to the manuscript are separated below according to sections. More generally, as suggested by reviewer #2, the language and phrasing has been revised throughout the manuscript. In addition, repetitive text has been either condensed or removed.

**Abstract**

Reviewer #2 pointed out that the abstract was rather long and had too many historical details. We wish, however, for the abstract to remain as first submitted. If this study was intended for an archaeobotanical journal we would condense the historical aspect. As this is an interdisciplinary study, with potential interest among readers from various research fields, we find that providing details on the historical context of the botanical finds to potentially be of interest when reading the abstract.

Word limit according to the journal is 300 words, our abstract is 268 word.

**Introduction**

Both reviewers commented on the *Introduction* section being rather lengthy. To address this, the *Introduction* section was reorganized. The two subheadings *Historical background* and *The Baltic preservative environment and Northern Europe maritime archaeobotanical comparanda* were merged and some text was removed. The research focus is more explicitly emphasized as well. Some historical data was removed as these aspects of the study is discussed in more detail in the *Discussion* section. As advised by reviewer #1, the part on preservation was placed in the *Introduction* to explain the site. Subheading *Archaeological background* was removed from the *Introduction*, and placed as a separate heading following the *Introduction*.

**Material and methods**

All material recovered were subfossil. Partial mineralization of plant remains was not observed.

**Results**

The absence of fig seeds in the samples is indeed, noteworthy. As pointed out by reviewer #2, seeds from figs is a common exotic food commodity in material from medieval towns. All samples were carefully analyzed and no botanical remains or species were overlooked. Fig seeds were simply not in the collected samples. However, future investigations of the shipwreck may present new species and complement the economic food plants presented in this study.

**Discussion**

The five food categories was revised as suggested by reviewer #2. Raspberry and blackberry has been placed under the category *Fruits and vegetables*, and the two nut species (almond, hazel) are placed in a separated category, *Nuts*. This reorganization is corrected throughout the manuscript.

Subheading *Oilseeds*. That flax may have had medicinal application has been commented.

Subheading *Medicinal plants*. As pointed out by reviewer #2, while henbane is an interesting plant, having served multiple purposes in medieval times, with only one seed found, it is somewhat unclear if it representing medicinal use or a ruderal weed. We therefore, as suggested by reviewer #2, shortened the discussion on henbane.

**Figures**

Figure 1. As suggested by reviewer #2, the map was edited to include locations of the main medieval towns along the Baltic coasts with archaeobotanical data discussed in the manuscript.

Figure 2. Resolution of figure 2 may have been affected by uploading the submission as a pdf when sent to reviewers.

**Tables**

Table 1. Plant categories was adjusted as suggested by reviewer #2. Berries are placed in *Fruits and berries*; *nuts* is a category on its own.

Table 2. To add sites and frequency of recorded number of seeds rather than presence and absence is a good point raised by reviewer #1. Based on the finds from *Gribshunden*, the purpose of the table was, however, for the reader to get a visual overview of the presence of these species found in the archaeological layers of medieval towns in Scandinavia and around the Baltic region. To include sites and seed frequency in the table is problematic for some species and geographical areas. As some original sources presenting archaeological data from the Baltic region is only reported or published in the native language of respective countries of the region, or reported in only domestic journals or books, we have had to use secondary literature for some archaeobotanical information. Thus, to list frequency for some species and only presence for others would be inconsistent. For this reason, we decided to keep presence and absence in Table 2 as originally submitted. Nevertheless, in the subsection *Economic plants from the Gribshunden shipwreck and archaeological comparanda* of the *Discussion* section, we discuss sites or town from where botanical remains of respective species have been found, and if botanical remains for the species is common or rare, or when possible, we mention frequency.

As dill, black mustard, caraway are common in cultural layers around Scandinavia and the Baltic region during the Middle Ages, these species were removed from table 2. Instead, we focus on the plants species found at the wreck site of Gribshunden that are rare and exotic in Scandinavia and the Baltic region.

**References**

References in the text have been double check to make sure that references are placed correctly.

Seven references were added to the manuscript:

Kamatou, GP, Vermaak, I, Viljoen, M, 2012. Eugenol - from the remote Maluku Island to the international market place: a review of a remarkable and versatile molecule. Molecules 2012, 17, 6953-6981

Etting V, Straetkvern K, Gregory D. Gribshunden. In: Nationalmuseets Arbejdsmark 2019. 2019. p. 102–13.

Franckenius, Johansson 1638(1659): Speculum botanicum. 2nd edition, Upsaliae

Thangaselvabal T., Gailce Leo Justin C., Leelamathi M. Black pepper (Piper nigrum L.) ‘the king of spices’ – A review. Agricultural reviews, 2008, vol 29, issue 2, 89-98.

Hooker J.D. 1886. The Flora of British India. L. Reeve and Co., London, pp. 78–95. 5.

Corbineau, R, Ruas, M-P, Barbier-Pain, D, Fornaciari, G, Dupont, H, Colleter, R. Plants and aromatics for embalming in Late Middle Ages and modern period: a synthesis of written sources and archaeobotanical data (France, Italy). Vegetation History and Archaeobotany, 2018, 27 (1), pp.151-164. <https://doi.org/10.1007/s00334-017-0620-4>

Golaris A. Saffron cultivation in Greece. In: Negbi M, editor. Saffron : Crocus sativus L. Amsterdam, Harwood Academic Publishers, 1999, p. 73-85

Two references were removed from the manuscript:

Lempiäinen M, Timonen T, Harju P, Alvik R. Underwater archaeobotany: plant and wood analyses from the Vrouw Maria, a 1771 shipwreck in the Finnish Baltic Sea. Veg Hist Archaeobot [Internet]. 2022;31(1):97–106. Available from: <https://doi.org/10.1007/s00334-021-00840-3>

Manders M, Kuijper W. Shipwrecks in Dutch Waters with Botanical Cargo or Victuals. In: Bakels C, Kamermans H, editors. Analecta Praehistorica Leidensia. Leiden: Leiden University; 2015. p. 141–72.

**Additional requirements outlined by the journal.**

1. Style requirements have been addresses for the title page.
2. Figure 1 and 2; format has been change from Pdf to tiff.
3. Table 1 and 2; reformatted to make them editable. In doing so, Table 2 had to be changed from landscape view to portrait view to meet journal requirements.
4. Reference list has been reviewed to make sure that it is complete and correct.
5. Repository information and permit numbers (incl. full name of the issuing authority, have been added to the section: Material and Method.
6. Funding information has been revised.
7. Copyright details has been added to the figure caption to Figs 1 and 2 in the manuscript and a Content Permission Form has been uploaded from illustrator Frida Nilsson at Lund University.
